# Ir‐CoO Active Centers Supported on Porous Al_2_O_3_ Nanosheets as Efficient and Durable Photo‐Thermal Catalysts for CO_2_ Conversion

**DOI:** 10.1002/advs.202300122

**Published:** 2023-03-17

**Authors:** Yunxiang Tang, Tingting Zhao, Hecheng Han, Zhengyi Yang, Jiurong Liu, Xiaodong Wen, Fenglong Wang

**Affiliations:** ^1^ Key Laboratory for Liquid‐Solid Structural Evolution and Processing of Materials Ministry of Education Shandong University Jinan 250061 P. R. China; ^2^ Shandong Technology Center of Nanodevices and Integration School of Microelectronics Shandong University Jinan 250100 P. R. China; ^3^ State Key Laboratory of Coal Conversion Institute of Coal Chemistry Chinese Academy of Sciences Taiyuan Shanxi 030001 P. R. China; ^4^ National Energy Center for Coal to Liquids Synfuels China Co., Ltd Huairou District Beijing 101400 P. R. China; ^5^ Shenzhen Research Institute of Shandong University Shenzhen Guangdong 518057 P. R. China

**Keywords:** “nanoheaters”, CO_2_ conversion, CoO carriers, Ir nanoparticles, photo‐thermal catalysis

## Abstract

Photo‐thermal catalytic CO_2_ hydrogenation is currently extensively studied as one of the most promising approaches for the conversion of CO_2_ into value‐added chemicals under mild conditions; however, achieving desirable conversion efficiency and target product selectivity remains challenging. Herein, the fabrication of Ir‐CoO/Al_2_O_3_ catalysts derived from Ir/CoAl LDH composites is reported for photo‐thermal CO_2_ methanation, which consist of Ir‐CoO ensembles as active centers that are evenly anchored on amorphous Al_2_O_3_ nanosheets. A CH_4_ production rate of 128.9 mmol g_cat⁻_^1^ h⁻^1^  is achieved at 250 °C under ambient pressure and visible light irradiation, outperforming most reported metal‐based catalysts. Mechanism studies based on density functional theory (DFT) calculations and numerical simulations reveal that the CoO nanoparticles function as photocatalysts to donate electrons for Ir nanoparticles and meanwhile act as “nanoheaters” to effectively elevate the local temperature around Ir active sites, thus promoting the adsorption, activation, and conversion of reactant molecules. In situ diffuse reflectance infrared Fourier transform spectroscopy (in situ DRIFTS) demonstrates that illumination also efficiently boosts the conversion of formate intermediates. The mechanism of dual functions of photothermal semiconductors as photocatalysts for electron donation and as nano‐heaters for local temperature enhancement provides new insight in the exploration for efficient photo‐thermal catalysts.

## Graphical abstract

This work prepares Ir‐CoO/Al_2_O_3_ catalysts to realize the highly efficient photo‐thermal catalytic CO_2_ methanation under mild conditions. The CoO nanoparticles function as photocatalysts to donate electrons for Ir nanoparticles and meanwhile act as “nanoheaters” to effectively elevate the local temperature around Ir active sites, thus promoting the adsorption, activation, and conversion of reactant molecules.

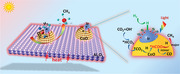

## 1 Introduction

Conversion of CO_2_ to useful chemicals offers a renewable approach to ameliorate the greenhouse effect, which emerges as one of the practical strategies to achieve net‐zero emission goals.^[^ ^1^ ^]^ Among the reported conversion approaches, reduction of CO_2_ to CH_4_ with H_2_ generated from renewable sources has been intensively explored, as CH_4_ is a clean fuel with high energy density and can be readily transported via natural gas pipelines.^[^ ^2^ ^]^ Compared with the solely light‐driven or the conventional thermal catalytic Sabatier reaction approaches, photo‐thermal catalytic processes where external heat and photo energy are coupled, overcome the low activity in sole solar‐driven catalytic processes and the harsh reaction conditions in thermal catalysis, thereby offering new avenues for efficient CO_2_ conversion under relatively mild conditions.^[^ ^3^ ^]^ In addition to the activation of reactants by external thermal energy, the photo‐excited charge carriers generated on semiconductor materials or the hot electrons produced on the surface of plasmonic metals could be injected into the antibonding orbitals of the reactants to activate the reactant molecules for redox reactions.^[^ ^4^ ^]^ It has been demonstrated that hot electrons on surface of Au nanoparticles effectively induced the dissociation of H_2_, which is a key step in many CO_2_ reduction reactions.^[^ ^5^ ^]^ As such, effective utilization of photo‐induced charge carriers is critical for enhancing the photochemical contribution in photo‐thermal catalytic process.^[^ ^6^ ^]^ To date, most reported photo‐thermal catalysts for gas‐phase heterogeneous catalysis are made of metal oxide‐based semiconductors decorated with metal nanoparticles, with inherent limitations including narrow light absorption spectrum and inferior separation efficiency of photo‐induced charge carriers.^[^ ^7^ ^]^ To overcome these obstacles, approaches including band gap engineering, introduction of defects, and internal electric field building have been studied to promote charge carrier generation and migration for metal/metal oxide catalysts.^[^ ^8^ ^]^

In addition to the promoted reactants adsorption/activation effect originating from the charge carriers, it is also found that the elevated local temperature around the active sites could dramatically accelerate the reaction kinetics. Recent advances have highlighted that localized photo‐to‐thermal conversion over photothermal materials, triggered via localized surface plasmon resonance (LSPR) or charge carrier relaxation, could dramatically accelerate the catalytic reaction by increasing the temperature of active sites.^[^ ^9^ ^]^ Zeng et al. encapsulated Au and Pt nanoparticles into ZIF‐8 to construct Au&Pt@ZIF catalysts, and found that the increased surface temperature of Pt active sites due to the LSPR effect of adjacent Au nanoparticles under light irradiation could enhance the photothermal CO_2_ hydrogenation for methanol production effectively.^[^ ^10^ ^]^ However, most of the investigated photothermal materials are noble metals such as Au and Ag, and their high cost and scarcity present obstacles to industrial application. To this end, the exploitation of inexpensive and naturally‐abundant nanomaterials that could replace noble metals as “nanoheaters” is of great significance. Based on these analyses, we believe that the fabrication of composited catalysts with both high charge carrier separation efficiency and photo‐to‐thermal conversion ability could be a promising approach to achieving efficient photo‐thermal conversion of CO_2_.

Herein, we report dramatically enhanced photo‐thermal CO_2_ methanation performances over Ir‐CoO/Al_2_O_3_ catalysts derived from Ir/CoAl LDH composites, where CoO carriers play dual critical roles in the hybrid by forming the intimate Ir‐CoO interfaces, expediting photo‐induced charge carrier generation and transportation, and functioning as “nanoheaters” to rapidly elevate the local temperature around the active sites, which benefit the adsorption and activation of CO_2_ molecules. The Al_2_O_3_ nanosheets with high thermal stability could effectively prohibit the agglomeration of tiny Ir‐CoO active centers during the reaction and thus assure the long durability of the composited catalysts. Thanks to the aforementioned merits, the optimized catalysts (0.16%Ir‐CoO/Al_2_O_3_) achieved an unprecedented CH_4_ production rate of 128.9 mmol g_cat⁻_^1^ h⁻^1^ with 92% selectivity and exceptional stability. By contrast, Ir/Al_2_O_3_ without CoO exhibits much lower catalytic activity under consistent conditions, highlighting the significance of CoO nanoparticles. To reveal the essence of intimate interaction between Ir and CoO, we impregnate Ir nanoparticles on the already prepared CoO/Al_2_O_3_ surface, and find that the produced Ir/CoO/Al_2_O_3_ catalysts show a CH_4_ production rate of 32 mmol g_cat⁻_^1^ h⁻^1^, which is much lower than that of Ir‐CoO/Al_2_O_3_. In situ diffuse reflectance infrared Fourier transform spectroscopy (In situ DRIFTS) measurements reveals that light irradiation brings no changes to the reaction pathways but efficiently boosts the rate‐determining step. To sum up, under light irradiation, the intimate interaction between Ir and CoO brings efficient charge generation and enhanced photothermal effect, which facilitated the adsorption and activation of reactant molecules and intermediate species and resulted in a higher CH_4_ production rate.

## 2 Results and Discussion

### 2.1 Characterizations

**Figure 1:**
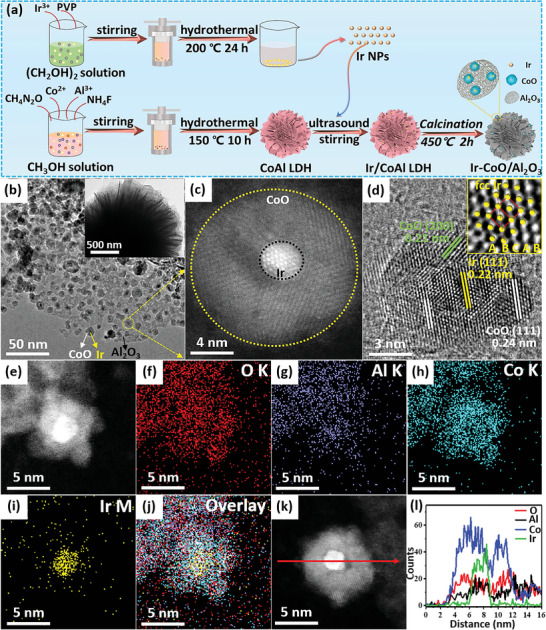
a) Schematic preparation procedures of Ir‐CoO/Al_2_O_3_ catalysts. b) TEM image, c) atomic‐resolution image, d) HRTEM image, e) aberration corrected HAADF‐STEM images and f–j) corresponding elemental mappings of 0.16% Ir‐CoO/Al_2_O_3_. (l) Compositional line scan profiles of O (red), Al (black), Co (blue) and Ir (green) of 0.16% Ir‐CoO/Al_2_O_3_ recorded along the arrow shown in the HAADF‐STEM image (k).

The as‐synthesized Ir nanoparticles (≈1.7 nm) were first supported on the surface of CoAl layered double hydroxide (LDH) to give the Ir/CoAl LDH composites, which were then calcined in a 5 vol.% H_2_/N_2_ stream for 2 h to produce the Ir‐CoO/Al_2_O_3_ catalysts (**Figure** **1**a). X‐ray diffraction (XRD) patterns confirmed the successful synthesis of Ir/CoAl LDH and the Ir‐CoO/Al_2_O_3_ hybrids, and the absence of Ir diffraction peaks in the patterns of composites could be attributed to the low content and high dispersity of the tiny Ir nanoparticles (Figure S2a, Supporting Information).^[^ ^11^ ^]^ Transmission electron microscopy (TEM) images manifested that Ir‐CoO/Al_2_O_3_ composites inherited the sheet‐like architecture of Ir/CoAl LDH precursors (Figure S4c, Supporting Information) and it should be noticed that the Ir nanoparticles were supported on CoO nanoparticles (≈10 nm) rather than on the porous Al_2_O_3_ substrate (Figure 1b,c and Figure S2c, Supporting Information). The corresponding high‐resolution TEM (HRTEM) image showed the lattice fringes with a distance of 0.22 and 0.24 nm which can be indexed to Ir (111) and CoO (111), respectively (Figure 1d). The typical atomic arrangement (ABCABC…) of Ir nanoparticles delineated the face‐centered‐cubic (fcc) structure (inset of Figure 1d). To further corroborate the microstructure, spherical aberration (Cs) corrected high‐angle annular dark‐field scanning TEM (HAADF‐STEM) images and corresponding energy‐dispersive X‐ray (EDX) elemental mappings (Figure 1e–j) were recorded and the results clearly showed that Ir elements were distributed on CoO, which confirmed the close contact between Ir and CoO. The detection of Al and O elements in supports implied that the supports were composed of amorphous Al_2_O_3_. In addition, the EDX line scan analysis provided further evidence that the Ir nanoparticles sat on CoO carriers (Figure 1k,l).

**Figure 2:**
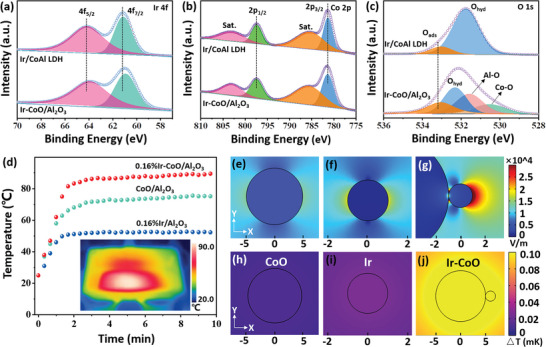
a) Ir 4f, b) Co 2p, and c) O 1s core‐level XPS spectra of 0.16% Ir/CoAl LDH and 0.16% Ir‐CoO/Al_2_O_3_. d) Temperature changes over 0.16% Ir/Al_2_O_3_, CoO/Al_2_O_3_ and 0.16% Ir‐CoO/Al_2_O_3_ catalysts (diluted in quartz sands) under visible light irradiation (420–780 nm, 2 W cm⁻^2^) and the IR image of 0.16% Ir‐CoO/Al_2_O_3_ under light irradiation (inset). (e–g) Induced electric field distributions and (h–j) temperature distributions on CoO, Ir nanoparticles and Ir‐CoO composites under light irradiation (420 nm, 2 W cm⁻^2^).

X‐ray photoelectron spectroscopy (XPS) measurements were carried out to probe the surface chemical properties and the interaction between the constituents of catalysts. In the core‐level Ir 4f spectrum of 0.16% Ir/CoAl LDH (**Figure** **2**a), two peaks located at 64.1 eV and 61.1 eV were assigned to Ir 4f_5/2_ and Ir 4f_7/2_ orbitals, suggesting that the loaded Ir existed in metallic state.^[^ ^12^ ^]^ The Co 2p spectrum could be deconvoluted into four peaks, and the two located at 797.4 eV and 781.3 eV corresponded to Co^2+^ species, while the other two at 803.5 eV and 786.7 eV could be mainly indexed to the shake‐up satellite peaks of Co^2+^.^[^ ^11^, ^13^ ^]^ It is worth noting that the Ir 4f_5/2_ and Ir 4f_7/2_ peaks of 0.16%Ir‐CoO/Al_2_O_3_ had shifted to lower binding energy regions with respect to 0.16%Ir/CoAl LDH, indicating the higher electron density on Ir species. This phenomenon could be attributed to the electron transfer from CoO to Ir, which agrees well with the fact that the work function of Ir and the electron affinity are CoO of 5.1 eV and 4.43 eV, respectively.^[^ ^14^ ^]^ The O 1s peaks of 0.16% Ir/CoAl LDH could be principally deconvoluted into two types of O species, which respectively corresponded to adsorbed oxygen (O_ads_) at 533.1 eV and hydroxyl species (O_hyd_) at 531.8 eV.^[^ ^15^ ^]^ Noticeably, two peaks at 530.4 eV and 531.5 eV observed on 0.16% Ir‐CoO/Al_2_O_3_ could be ascribed to metal‐oxygen bonds (Co‐O and Al‐O), which was in line with the XRD result and demonstrated the formation of metal oxides.^[^ ^15^ ^]^ In addition, XPS spectra under light irradiation (visible irradiation provided with Xenon lamp) were further obtained to study the direction of electron transfer in the Ir‐CoO/Al_2_O_3_ composites (Figure S5, Supporting Information). Under light irradiation, the binding energy of Ir 4f shift to lower energy level, while the binding energy of Co 2p shift to higher energy level, suggesting that the photogenerated electrons transfer from CoO to Ir nanoparticles. These results validated the intimate interaction and electron transfer between Ir nanoparticles and CoO in Ir‐CoO/Al_2_O_3_ composites.

As aforementioned, the photothermal effect has been demonstrated to play significant roles in promoting chemical conversion by effectively increasing the local temperature around the active sites. Therefore, the photo‐to‐thermal conversion ability of Ir/Al_2_O_3_, CoO/Al_2_O_3_, and Ir‐CoO/Al_2_O_3_ catalysts was studied (Figure 2d). Under light irradiation (420‐780 nm, 2 W cm⁻^2^), the surface temperature of the three catalysts (50 mg of the catalysts were diluted into 1.2 g of quartz sands) increased rapidly and reached the plateaus after ≈3 min. The peaked surface temperature of 0.16% Ir/Al_2_O_3_ and CoO/Al_2_O_3_ were 53 and 75 °C, respectively. Notably, the highest surface temperature of 0.16% Ir‐CoO/Al_2_O_3_ reached 90 °C, which was 37 and 15 °C higher than that of 0.16% Ir/Al_2_O_3_ and CoO/Al_2_O_3_, respectively. To gain insight into the enhancement mechanism of photothermal effect of Ir‐CoO/Al_2_O_3_, the induced electric field distributions of CoO, Ir nanoparticles and Ir‐CoO in the presence of 420 nm illumination were simulated by COMSOL Multiphysics based on finite element methods. As shown in Figure 2e, the sole CoO exhibited extremely weak electric field intensity under light irradiation and Ir nanoparticle showed some electric field strength due to the LSPR effect (Figure 2f).^[^ ^16^ ^]^ Surprisingly, when the Ir nanoparticle were in close contact with CoO, an extremely intense localized electromagnetic field was produced at the interface, resulting in near‐field enhancement due to the LSPR effect near the semiconductor surface (Figure 2g).^[^ ^17^ ^]^ High field intensity also suggested more charge carrier generation and transportation,^[^ ^18^ ^]^ which is consistent with the observation that 0.16% Ir‐CoO/Al_2_O_3_ exhibited much higher photocurrent response compared with CoO/Al_2_O_3_ (Figure S6, Supporting Information) and this benefits the activation of reactant molecules upon acceptance electrons in the antibonding orbitals.^[^ ^19^ ^]^ Moreover, the generated heat arising from the decay process of the enhanced localized electromagnetic field would also result in the dramatic temperature increase around the active sites, which would effectively reduce the activation energy of the reactant molecules as well.^[^ ^20^ ^]^ To this end, the steady temperature distributions of related samples were also simulated (Figure 2h–j). The result indicates that under light irradiation, the generated heat originating from relaxation of the induced high electric field gives rise to significant temperature increase at the interfaces and the thermal energy is energetically transferred to the Ir nanoparticles due to the higher thermal conductivity of Ir. Therefore, the CoO nanoparticles at the Ir‐CoO interfaces acted as photocatalysts to provide charge carriers and as “nanoheaters” to increase the local temperature rapidly around the Ir active sites, which led to the efficient activation of reactants.

### 2.2 Photo‐Thermal Catalytic Performance

**Figure 3:**
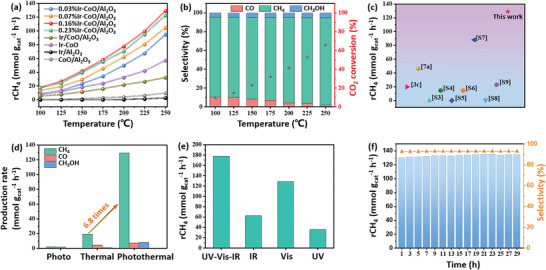
a) Effect of temperature on CH_4_ yield rate over Ir‐CoO, Ir/Al_2_O_3_, CoO/Al_2_O_3_, Ir‐CoO/Al_2_O_3_ and Ir/CoO/Al_2_O_3_ catalysts. b) Product selectivity and CO_2_ conversion of 0.16%Ir‐CoO/Al_2_O_3_ catalyst under light irradiation at different temperatures. c) Comparison of the CH_4_ production rate of our work with those of representative previous studies (numbers in the figure are their corresponding reference numbers). d) Production rate of CH_4_, CO and CH_3_OH of 0.16% Ir‐CoO/Al_2_O_3_ catalyst under different conditions. e) CH_4_ yield rate over 0.16% Ir‐CoO/Al_2_O_3_ catalyst under various irradiation conditions with constant light intensity. f) CH_4_ yield rate and selectivity of 0.16% Ir‐CoO/Al_2_O_3_ catalyst during long‐time reaction at 250 °C under light irradiation. Reaction conditions: 100–250 °C, 0.1 MPa, 2 W cm⁻^2^ in light intensity, GHSV = 24000 cm^3^ h⁻^1^ g_cat⁻_^1^, and H_2_:CO_2_ = 4:1.

The photo‐thermal CO_2_ hydrogenation reaction was used as the probe reaction to evaluate the catalytic performance of Ir/Al_2_O_3_, Ir‐CoO, CoO/Al_2_O_3_, Ir‐CoO/Al_2_O_3_, and Ir/CoO/Al_2_O_3_. To avoid the flow choking caused by the agglomeration of nanocatalysts during reaction process, we packed the reactor with mixture of 50 mg catalysts and 1.2 g quartz sands (≈0.42 mm in diameter). As shown in **Figure** **3**a, the composition of the catalysts and the temperature greatly affected the catalytic performances. All catalysts exhibited growing catalytic activities with the increase of reaction temperature and the catalytic behaviors of different catalysts were compared at 250 °C. The CoO/Al_2_O_3_ catalyst showed negligible catalytic activity with a CH_4_ production rate of 6.85 mmol g_cat⁻_^1^ h⁻^1^ at 250 °C under light irradiation, indicating the necessity of catalytically active Ir nanoparticles. Compared with CoO/Al_2_O_3_, all the Ir‐CoO/Al_2_O_3_ catalysts showed significantly enhanced catalytic activity, and the CH_4_ production rate displayed a volcano‐like tendency with respect to the Ir content. The highest CH_4_ production rate of 128.9 mmol g_cat⁻_^1^ h⁻^1^ (80.6 mol g_Ir⁻_^1^ h⁻^1^) was obtained at 250 °C under light irradiation (external heat is about 200 °C, Figure S7, Supporting Information) when catalyzed by 0.16% Ir‐CoO/Al_2_O_3_ (Figure 3a), significantly outperforming other reported metal‐based catalysts (Figure 3c and Table S2, Supporting Information). Moreover, it was found that the selective production of CH_4_ was enhanced with the rise of temperatures and the 0.16% Ir‐CoO/Al_2_O_3_ catalyst exhibited a prominent selectivity of 92% to CH_4_, corresponding to a CO_2_ conversion of 65.5% at 250 °C (Figure 3b). In comparison with Ir‐CoO/Al_2_O_3_, the Ir/Al_2_O_3_ and Ir‐CoO catalysts showed substantially lower catalytic activity of 4.51 mmol g_cat⁻_^1^ h⁻^1^ and 56.9 mmol g_cat⁻_^1^ h⁻^1^, respectively, indicating that the Ir and CoO interaction and the stabilizing effect of Al_2_O_3_ supports play significant roles in the CO_2_ hydrogenation process. This assumption could also be verified by the catalytic performance and structure evolution of 0.16% Ir/CoAl LDH catalyst (Figure S8, Supporting Information). In addition, to further reveal the significance of the Ir and CoO interaction on the catalytic performance, we impregnated Ir nanoparticles on CoO/Al_2_O_3_ surface to produce the Ir/CoO/Al_2_O_3_ catalyst and compared its activity with that of Ir‐CoO/Al_2_O_3_. To our surprise, the Ir/CoO/Al_2_O_3_ catalyst exhibited only mediocre CH_4_ production rate of 32 mmol g_cat⁻_^1^ h⁻^1^ under the constant condition (≈1/4 of that obtained over Ir‐CoO/Al_2_O_3_, Figure 3a), indicating that the efficient fabrication of the Ir‐CoO interface was critical in achieving excellent catalytic performance. Furthermore, we explored the structure and catalytic performance of the composites with different metal nanoparticles (Ru, Rh, Pt and Pd), and the corresponding XRD patterns, TEM images and catalytic activity are shown in Figure S9 (Supporting Information). These results further revealed that the intimate interaction between metal nanoparticles and CoO were critical for the enhanced catalytic performance.

To investigate the effect of light on catalytic activity, the CH_4_ production rates under thermal catalytic (in dark), solely light‐driven and photo‐thermal conditions were compared and the results are shown in Figure 3d. At 250 °C in dark, a CH_4_ production rate of 18.8 mmol g_cat⁻_^1^ h⁻^1^ was achieved over 0.16% Ir‐CoO/Al_2_O_3_ catalyst and a much lower value of 1.9 mmol g_cat⁻_^1^ h⁻^1^ was obtained in the presence of sole light irradiation, suggesting that the photo‐thermal catalytic CO_2_ hydrogenation was dominantly a thermal‐driven process. Strikingly, ≈sevenfold enhancement of CH_4_ production rate (128.9 mmol g_cat⁻_^1^ h⁻^1^) was observed in photo‐thermal process compared with the thermal catalytic process, demonstrating that light irradiation could significantly enhance the thermal catalytic reaction (Figure 3d and Figure S10a, Supporting Information). The corresponding Arrhenius plot showed one‐stage linear plots with activation energies of 24.77 kJ mol⁻^1^ in dark and 21.57 kJ mol⁻^1^ under light irradiation, elucidating that illumination decreased the apparent activation energy (Figure S10b, Supporting Information) and it attributed to the improved activation of CO_2_ molecules and the formate intermediate. Additionally, to explore the effect of light wavelength ranges on the catalytic activity, a series of tests were performed over 0.16% Ir‐CoO/Al_2_O_3_ catalyst at 250 °C under Xe lamp irradiation with different filters (the light intensity was fixed at ≈2W cm⁻^2^). As shown in Figure 3e, the CH_4_ production rate under infrared (62.4 mmol g_cat⁻_^1^ h⁻^1^) and UV light (35.7 mmol g_cat⁻_^1^ h⁻^1^) irradiation was much lower than that under visible light (128.9 mmol g_cat⁻_^1^ h⁻^1^) irradiation, indicating that the plasmonic absorption in the visible light region of Ir‐CoO/Al_2_O_3_ catalysts greatly contributed to the high activity. To further highlight the significance of light source, the catalytic activity of 0.16% Ir‐CoO/Al_2_O_3_ was also evaluated under full spectrum irradiation and a much higher CH_4_ production rate of 177.9 mmol g_cat⁻_^1^ h⁻^1^ was achieved, far exceeding the performance under visible irradiation. This could be attributed to the greatly increased photo‐induced electrons generated due to the strong absorption across the UV‐Vis‐NIR region of Ir‐CoO/Al_2_O_3_ catalysts. These results clearly showed that the external heat and light irradiation synergistically enhanced CO_2_ hydrogenation performance, achieving a much higher CH_4_ production rate than the sum under sole light irradiation and sole thermal catalytic condition. However, it is still challenging to quantify the individual contribution from light irradiation and external heating process.

To assess the industrial application potential of Ir‐CoO/Al_2_O_3_ catalysts, a 30 h long‐term stability test was conducted at 250 °C under light irradiation in the flow reactor. As shown in Figure 3f, no noticeable decrease in CH_4_ production rate was detected during 30 h of catalytic reactions, indicating that the Ir‐CoO/Al_2_O_3_ catalysts were highly stable. Besides, the stability of catalysts in dark (250 °C) was also investigated (Figure S11, Supporting Information). It was found that the production rate and selectivity of CH_4_ decreased with time, which suggested that the light irradiation benefited the catalyst stability. In addition, XRD pattern, TEM image and XPS spectra of the spent 0.16% Ir‐CoO/Al_2_O_3_ were recorded to examine the structure evolution and the results demonstrated the robust stability of the composites in the CO_2_ hydrogenation reaction process (Figures S12 and S13, Supporting Information). To validate that the product originated from CO_2_ hydrogenation, we conducted the study with different reaction gases (Figure S14). Obviously, no products were detected in the H_2_+Ar and CO_2_+Ar atmosphere, demonstrating that the produced CO, CH_4_ and CH_3_OH should be attributed to CO_2_ hydrogenation process.

### 2.3 Proposed Reaction Mechanisms of CO_2_ Methanation

**Figure 4:**
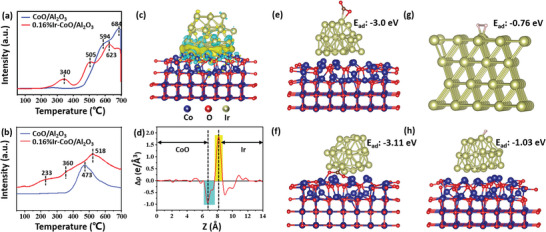
a) H_2_‐TPR and b) CO_2_‐TPD profiles of CoO/Al_2_O_3_ and 0.16% Ir‐CoO/Al_2_O_3_. c) Side view of the charge density difference and d) planar‐averaged electron density difference ∆*ρ* (z) for Ir‐CoO, and the cyan and yellow areas represent electron depletion and accumulation, respectively. Optimized geometries of CO_2_ molecules adsorbed on e) Ir surface and at f) Ir‐CoO interface. Optimized geometries of H_2_ molecules adsorbed on g) Ir and h) Ir‐CoO. The blue, red, yellowish‐green, grey and brown spheres represent Co, O, Ir, H, and C atoms, respectively.

To investigate the reduction behavior of catalysts and the interaction between Ir species and support, H_2_ temperature‐programmed reduction (H_2_‐TPR) measurements were carried out. As depicted in **Figure** **4**a, the CoO/Al_2_O_3_ showed two broad peaks at 594 and 684 °C, which could be attributed to the reduction of CoO to metallic Co and the reduction of cobalt aluminates species, respectively.^[^ ^21^ ^]^ For 0.16% Ir‐CoO/Al_2_O_3_, the peak at 340 °C was assigned to the reduction of the IrO_2_ species that strongly interacted with CoO and the other two peaks at high temperature regions corresponded to the reduction of CoO and cobalt aluminates species.^[^ ^22^ ^]^ Notably, the addition of Ir led to the decrease in the reduction temperature of CoO and cobalt aluminates species toward 505 and 623 °C, respectively, which could be ascribed to the efficient dissociation and spillover of H_2_, elucidating that H_2_ molecules could be easily dissociated on Ir‐CoO/Al_2_O_3_ to participate in the hydrogenation reaction. In addition, the CO_2_ adsorption properties of CoO/Al_2_O_3_ and 0.16% Ir‐CoO/Al_2_O_3_ catalysts were studied using CO_2_ temperature‐programmed desorption (CO_2_‐TPD) and the results are shown in Figure 4b. The desorption peak at around 473 °C on CoO/Al_2_O_3_ catalyst was related to the chemically adsorbed CO_2_ molecules.^[^ ^15a^ ^]^ The 0.16% Ir‐CoO/Al_2_O_3_ catalyst exhibited three desorption peaks at 233, 360, and 518 °C, which could be assigned to the weakly and strongly adsorbed CO_2_ molecules on the surface of 0.16% Ir‐CoO/Al_2_O_3_ catalyst. Apparently, compared with CoO/Al_2_O_3_, the 0.16%Ir‐CoO/Al_2_O_3_ catalyst showed stronger CO_2_ adsorption capacity when the temperature was lower than 400 °C, indicating that the Ir species could promote adsorption of CO_2_ on the catalyst. Generally, valid CO_2_ adsorption is one of the prerequisites for high CO_2_ conversion efficiency.^[^ ^2b^ ^]^ This may be a credit from the formation of Ir‐CoO interface, which promotes the adsorption of CO_2_.

To understand the effect of charge transfer between Ir nanoparticles and CoO on the Sabatier reaction kinetics, density functional theory (DFT) calculations were conducted. As revealed by the charge difference distribution of Ir‐CoO in Figure 4c, electron transfer from CoO to Ir nanoparticles is energetically favored through the intimate interfaces, which is consistent with the XPS results. In addition, the planar‐averaged charge density difference along the Z direction displays the change of charge density, which enables us to observe the surface charge of Ir and CoO directly (Figure 4d). The positive values and negative values represent electron accumulation and depletion, respectively.^[^ ^23^ ^]^ This result unveils that the CoO close to the interface is positively charged, whereas the Ir near the interface is negatively charged due to the electron transfer from CoO to Ir.

To further clarify the CO_2_ adsorption sites on the catalysts surface, the CO_2_ adsorption behavior on Ir surface and at Ir‐CoO interface is investigated by DFT calculations. The optimized geometries of CO_2_ adsorbed on Ir surface and at Ir‐CoO interface are exhibited in Figure 4e,f. On the Ir surface, the C‐O bond lengths are increased from 1.16 Å to 1.22 Å and 1.33 Å, and the bond angle decrease from 180° to 142.4°; while at the Ir‐CoO interface, the C‐O bond lengths increase to 1.28 Å and 1.29 Å, and the bond angle decreases to 127.2°, indicating easier dissociation of CO_2_ molecules at the Ir‐CoO interface.^[^ ^24^ ^]^ Besides, the adsorption energy (E_ads_) of CO_2_ on the Ir surface and at Ir‐CoO surface are ‐3.0 and ‐3.11 eV, respectively, suggesting that the CO_2_ is more easily adsorbed at the Ir‐CoO interface. Therefore, the Ir‐CoO interface is considered as the most active adsorption site of CO_2_ molecules. In addition, to shed light on the effect of the photo‐induced electrons on the adsorption of reactants, the adsorption behavior of H_2_ molecules on the surface of Ir nanoparticles was also explored by DFT calculations. We simulated the neutral and negatively charged Ir surface by constructing the geometries of Ir(111) and Ir(111)‐CoO, denoted as [Ir(111)] and [Ir(111)]^–^, respectively. Figure 4g,h shows the optimized geometries of H_2_ adsorbed [Ir(111)] and [Ir(111)]^–^ surfaces, and the corresponding bond lengths and adsorption energy are also simulated (Table S3, Supporting Information), and again the results indicated that H_2_ is more easily adsorbed on the [Ir(111)]^–^ surface. The σ*antibonding orbitals of H_2_ molecules could then accept electrons from the negatively charged Ir surface, and this process would enable the dissociation of adsorbed H_2_ into H species, which would readily react with the adsorbed CO_2_ molecules to initiate the hydrogenation process.^[^ ^25^ ^]^

**Figure 5:**
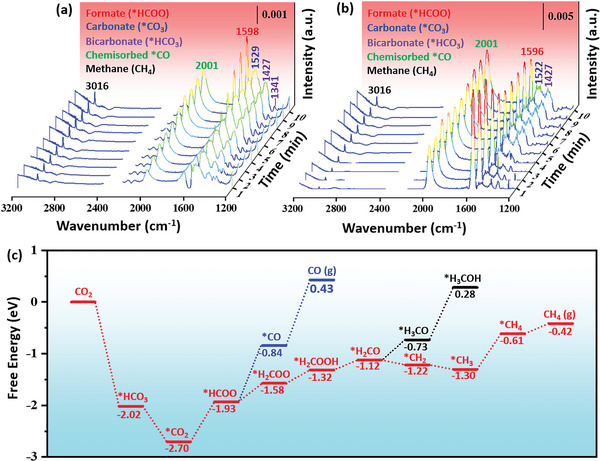
In situ DRIFTS spectra of 0.16% Ir‐CoO/Al_2_O_3_ catalyst in dark (a) and under light irradiation (b) at 300 °C for 10 min. c) Relative free energy changes in potential pathways for CO_2_ hydrogenation to CO, CH_4_, and CH_3_OH.

To track the reaction intermediates during the photo‐thermal CO_2_ methanation process over Ir‐CoO/Al_2_O_3_, in situ DRIFTS measurements were carried out. As shown in Figure S15 (Supporting Information), when CO_2_ and H_2_ were introduced into the reactor at room temperature, several vibrational peaks could be observed at 1385, 1530 and 1628 cm⁻^1^. The bands at 1385/1628 and 1529 cm⁻^1^ were ascribed to the formation of bicarbonate (*HCO_3_) and carbonates (*CO_3_) species, respectively,^[^ ^26^ ^]^ indicating that CO_2_ was first adsorbed and existed in the form of *HCO_3_ and *CO_3_. Noticeably, these bands of *HCO_3_ disappeared as the temperature reached 200 °C, whereas vibrations peaks at 1362 and 1583 cm⁻^1^ corresponding to the formate species (*HCOO) appeared,^[^ ^26^, ^27^ ^]^ suggesting that *HCO_3_ was transformed into *HCOO. As the temperature further increased to 300 °C, two new peaks were observed at 1341 and 1427 cm⁻^1^, which were ascribed to *HCO_3_ species,^[^ ^28^ ^]^ hinting that high temperature was favorable for CO_2_ activation (**Figure** **5**a). Notably, compared to the spectra at 200 °C, the band at 1583 cm⁻^1^ of *HCOO exhibited a small shift to the higher frequency region by 15 cm⁻^1^ at 300 °C. The increase in vibrational frequency indicates that the adsorption of *HCOO species on catalyst surface was weakened.^[^ ^27b^ ^]^ The band at 2001 cm⁻^1^ was ascribed to the linear CO adsorption on Ir^0^,^[^ ^29^ ^]^ indicating that *CO species was formed in the process of CO_2_ methanation. Additionally, a sharp band at 3016 cm⁻^1^ was observed corresponding to the C‐H stretching vibrations of CH_4_.^[^ ^30^ ^]^ The intensity of these peaks increased over time and peaked after 10 min. Upon light irradiation, the peak intensity of *HCOO species gradually decreased while the CH_4_ signal gradually increased, indicating that the *HCOO species was the crucial intermediate in the conversion of CO_2_ to CH_4_ (Figure 5b). This result revealed that light irradiation could promote the conversion of *HCOO intermediate to CH_4_, leading to a decreased coverage of *HCOO species on the catalyst surface. In addition, the *CO amount presented a remarkably increasing trend over time, indicating that *CO made no contribution to the generation of CH_4_. Therefore, the in situ DRIFTS results demonstrated that CO_2_ methanation on the Ir‐CoO/Al_2_O_3_ catalyst followed the formate pathway, which is in line with previous works.^[^ ^26b^ ^]^

Furthermore, DFT calculations were carried out based on the models of Ir supported on the CoO (200) slabs to illustrate the reaction pathway, and the change of free energy was indicated for each elementary step (Figure 5c). The optimized configurations of the involved intermediates are shown in Figure S16 (Supporting Information). As shown in Figure 5c, the formed *CO_2_ can be hydrogenated to *HCOO with an energy change of 0.77 eV. The further hydrogenation of *HCOO to *H_2_COO (energy change: 0.35 eV) is thermodynamically more favorable than the dissociation of *HCOO to *CO (energy change: 1.09 eV), which is consistent with the fact that the selectivity of CO is relatively low. The generated *H_2_COO is further hydrogenated to *H_2_COOH, with an energy change of 0.26 eV. Afterward, the *H_2_COOH dissociates into *H_2_CO and *OH via the direct C‐O bond cleavage pathway. Notably, the further dissociation of *H_2_CO to *CH_2_ is evidently more favorable than the hydrogenation of *H_2_CO to *H_3_CO, and therefore, *CH_2_ is hydrogenated to CH_4_. Namely, the *H_2_CO species favor dissociation energetically rather than hydrogenation, resulting in lower CH_3_OH selectivity and higher selectivity for the targeted CH_4_.

**Figure 6:**
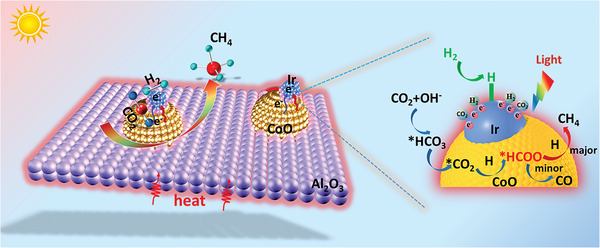
Proposed reaction mechanism for photo‐thermal catalytic Sabatier reaction process over Ir‐CoO/Al_2_O_3_.

Based on the detected intermediate species, the possible reaction mechanisms of photo‐thermal CO_2_ methanation on Ir‐CoO/Al_2_O_3_ catalysts are proposed (**Figure** **6**). First, adsorbed CO_2_ is converted into *HCO_3_ species, which then transform into the CO_2_*.^[^ ^26b^ ^]^ Subsequently, *CO_2_ accepts the H atoms generated via the dissociation of H_2_ on Ir nanoparticles to form *HCOO intermediates. According to the results of DFT calculations, the *HCOO species could be hydrogenated to *HCOOH and further hydrogenated to *H_2_COOH. Then *H_2_COOH undergoes consecutive decomposition to produce *CH_2_, and *CH_2_ was eventually hydrogenated to CH_4_. In addition, a small portion of the *HCOO intermediates disassociate into *CO and H_2_O, and the *CO is not converted to CH_4_, but desorbed to produce CO gas. Furthermore, light irradiation does not change the reaction pathway, but promotes the adsorption activation and conversion of *HCOO intermediates, which is the rate‐determining step for the CO_2_ hydrogenation to CH_4_, thus effectively facilitating the CH_4_ formation. Combined with the aforementioned analysis based on DFT calculations and finite element methods based numerical simulations, in the thermal‐driven CO_2_ hydrogenation process, light irradiation results in the improved adsorption and activation of the reactants molecules by inducing the efficient utilization of charge carriers and enhanced photothermal effect, and simultaneously promotes the conversion of *HCOO intermediates to CH_4_, thus synergistically boosting the overall CO_2_ methanation efficiency in the global heating reaction system.

## 3 Conclusion

In summary, we have prepared Ir‐CoO/Al_2_O_3_ catalysts to realize the highly efficient photo‐thermal catalytic CO_2_ methanation under mild conditions. Our comprehensive investigation demonstrated that the significantly enhanced catalytic performance was attributed to the intimate interaction between Ir and CoO and the stabilizing effect of the Al_2_O_3_ supports. The formation of Ir‐CoO interfaces resulted in strong localized electric field to accelerate charge carrier generation and migration under illumination. DFT calculations revealed that the electron transfer from CoO to Ir nanoparticles favored the H_2_ dissociation. In addition, CoO also acted as “nanoheaters” to elevate the local temperature of active sites to accelerate the reaction kinetics, which further enhanced the catalytic performance. In situ DRIFTS confirmed that the CO_2_ hydrogenation on Ir‐CoO/Al_2_O_3_ catalysts followed the formate pathway and light irradiation efficiently boosted the activation and conversion of *HCOO intermediates. Owing to these contributions from light irradiation, a high CH_4_ production rate of 128.9 mmol g_cat⁻_^1^ h⁻^1^ (80.6 mol g_Ir⁻_^1^ h⁻^1^) was achieved over 0.16% Ir‐CoO/Al_2_O_3_ catalyst at 250 °C under ambient pressure, outperforming most of the reported metal‐based catalysts. This work provides in‐depth understanding on the synergistic effects in photo‐thermal catalytic process and proposes a novel catalyst for the efficient conversion of CO_2_ under mild conditions.

## Conflict of Interest

The authors declare no conflict of interest.

## Supporting information

Supporting Information

## Data Availability

The data that support the findings of this study are available from the corresponding author upon reasonable request.
