# Supplementary material for: Ir‐CoO Active Centers Supported on Porous Al2O3 Nanosheets as Efficient and Durable Photo‐Thermal Catalysts for CO2 Conversion
Source: Adv Sci (Weinh). 2023 Mar 17;10(15):2300122. doi: 10.1002/advs.202300122 (PMC10214215; doi:10.1002/advs.202300122)
Supplement: Supplementary file 1 — Supporting Information [file ADVS-10-2300122-s001.pdf]

ADVANCED SCIENCE
Open Access

# Supporting Information

for *Adv. Sci.*, DOI 10.1002/advs.202300122

## Ir-CoO Active Centers Supported on Porous $Al_2O_3$ Nanosheets as Efficient and Durable Photo-Thermal Catalysts for $CO_2$ Conversion

*Yunxiang Tang, Tingting Zhao, Hecheng Han, Zhengyi Yang, Jiurong Liu, Xiaodong Wen* and Fenglong Wang**

**Supporting Information**

# Ir-CoO active centers supported on porous $Al_2O_3$ nanosheets as efficient and durable photo-thermal catalysts for $CO_2$ conversion

Yunxiang Tang[a], Tingting Zhao[a], Hecheng Han[b], Zhengyi Yang[a], Jiurong Liu[a], Xiaodong Wen[c, d, *], Fenglong Wang[a, e, *]

[a] Key Laboratory for Liquid-Solid Structural Evolution and Processing of Materials Ministry of Education, Shandong University, Jinan, 250061, P. R. China

[b] Shandong Technology Center of Nanodevices and Integration, School of Microelectronics, Shandong University, Jinan, 250100, P. R. China

[c] State Key Laboratory of Coal Conversion, Institute of Coal Chemistry, Chinese Academy of Sciences, Taiyuan, Shanxi, 030001, P. R. China

[d] National Energy Center for Coal to Liquids, Synfuels China Co., Ltd, Huairou District, Beijing, 101400, P. R. China

[e] Shenzhen Research Institute of Shandong University, Shenzhen, Guangdong, 518057, P. R. China

***Corresponding Author's Emails:**

wxd@sxicc.ac.cn

fenglong.wang@sdu.edu.cn (F.L.W)

## Experimental section:

### Materials

Iridium acetate ($Ir(OAc)_3$), ruthenium chloride hydrate ($RuCl_3$ $xH_2O$), rhodium bromide dihydrate ($RhBr_3$ $2H_2O$), chloroplatinic acid hexahydrate ($H_2PtCl_6{\cdot}6H_2O$), palladium chloride ($PdCl_2$) and polyvinylpyrrolidone (PVP) were obtained from Shanghai Macklin Biochemical Co., Ltd. Cobalt nitrate hexahydrate ($Co(NO_3)_2$ $6H_2O$), aluminum nitrate nonahydrate ($Al(NO_3)_3$ $9H_2O$), urea and ammonium fluoride ($NH_4F$) were provided from Shanghai Aladdin Biochemical Technology Co., Ltd. All the chemicals were of analytical grade and used are received.

### Synthesis of Ir nanoparticles

Ir nanoparticles were synthesized *via* a solvothermal method employing $Ir(OAc)_3$ as precursor and PVP (M.W.: ~40000) as the stabilizing agent. Typically, 0.02 mmol of $Ir(OAc)_3$ and 100 mg of PVP were dissolved into 15 mL of ethylene glycol forming the precursor solution, which was then transferred into a 25 mL stainless steel autoclave and heated at 200 ℃ for 24 h to produce the Ir nanoparticles.

### Synthesis of CoAl layered double hydroxide (LDH)

Typically, 2.1 g of $Co(NO_3)_2$ $6H_2O$, 1.35 g of $Al(NO_3)_3$ $9H_2O$, 1.2 g of urea and 0.18 g of $NH_4F$ were dissolved into 50 mL methanol under magnetic stirring. Then the solution was transferred into a 100 mL stainless steel autoclave and heated at 150 ℃ for 12 h. After being washed for several times with ethanol, the obtained LDH

powders were dried at 70 ℃ for further use.

**Synthesis of Ir/CoAl LDH**

Ir nanoparticles were deposited onto CoAl LDH by a simple impregnation method. In brief, 200 mg of the obtained CoAl LDH was dispersed in 25 mL of ethanol, and then a certain amount of Ir nanoparticles suspension was introduced into the mixture under sonication. Subsequently, the mixture was stirred for 6 h at room temperature. The final products were collected by centrifugation and dried at 70 ℃ for 12 h. By changing the volume of Ir nanoparticles suspension, Ir/CoAl LDH catalysts with different Ir contents were prepared and denoted as 0.03%Ir/CoAl LDH, 0.07%Ir/CoAl LDH, 0.16%Ir/CoAl LDH and 0.23%Ir/CoAl LDH according to the Ir content in weight percentage, respectively. The Ir contents (wt.%) in the Ir/CoAl LDH were confirmed by inductively coupled plasma optical emission spectrometry (ICP-OES, Agilent 7800, Table S1).

**Synthesis of Ir-CoO/$Al_2O_3$**

The Ir-CoO/$Al_2O_3$ were obtained by calcining the Ir/CoAl LDH in 5 vol.% $H_2/N_2$ stream at 450 ℃ for 2 h with the ramping rate of 3 ℃/min. The obtained Ir-CoO/$Al_2O_3$ hybrids were denoted as 0.03%Ir-CoO/$Al_2O_3$, 0.07%Ir-CoO/$Al_2O_3$, 0.16%Ir-CoO/$Al_2O_3$ and 0.23%Ir-CoO/$Al_2O_3$ according to the Ir contents in Ir/CoAl LDH. CoO/$Al_2O_3$ was also prepared using the same method without Ir loading.

**Synthesis of Ir-CoO and Ir/$Al_2O_3$**

The synthetic procedures for Ir-CoO and Ir/$Al_2O_3$ were similar to Ir-CoO/$Al_2O_3$, except for the introduction of Al or Co precursor in the LDH preparation process.

**Synthesis of Ir/CoO/$Al_2O_3$**

The as-synthesized Ir nanoparticles suspension was added dropwise to the CoO/$Al_2O_3$ ethanol suspension and stirred for 6 h. Then the precipitates were collected by centrifugation and dried in an oven at 70 ℃ for 12 h. The sample was described as Ir/CoO/$Al_2O_3$.

**Synthesis of Ru Nanoparticles**

Typically, 100 mg of PVP (M.W.: ~40000) was dissolved in 8 mL of ethylene glycol, and then 2 mL of $RuCl_3$ aqueous solution (50 mmol/L) was added dropwise into the above PVP solution. The mixture was then heated at 190 ℃ for 15 min in an oil bath to produce the Ru nanoparticles.

**Synthesis of Rh Nanoparticles**

Typically, 29.9 mg of $RhBr_3$, 193 mg of PVP (M.W.: ~40000) and 10 mL of ethylene glycol were added into a vial under magnetic stirring for 1 h. Then the resulted homogeneous mixture was heated at 140 ℃ for 90 min.

**Synthesis of Pt Nanoparticles**

Typically, 100 mg of PVP (M.W.: ~40000) was dissolved in 10 mL of ethylene glycol, and then 0.1 mL of $H_2PtCl_6·6H_2O$ aqueous solution (0.2 mol/L) was injected into the above mixture to give the precursor solution, which was then heated at 180 ℃ for 20 min.

**Synthesis of Pd Nanoparticles**

Typically, 50 mg of PVP (M.W.: ~40000), 170 mg of KI and 36 mg of $PdCl_2$ were dissolved in 5 mL of formamide forming the precursor solution, which was then maintained at 120 for 60 min to produce Pd nanoparticles.

**Synthesis of Ru, Rh, Pt and Pd nanoparticles loaded M-CoO/$Al_2O_3$ (M=Ru, Rh, Pt or Pd)**

The Ru, Rh, Pt and Pd nanoparticles loaded CoAl LDH were synthesized following a similar procedure to Ir/CoAl LDH and the obtained composites were denoted as Ru/CoAl LDH, Rh/CoAl LDH, Pt/CoAl LDH and Pd/CoAl LDH. The M-CoO/$Al_2O_3$ composited catalysts including Ru-CoO/$Al_2O_3$, Rh-CoO/$Al_2O_3$, Pt-CoO/$Al_2O_3$, and Pd-Co/$Al_2O_3$ were obtained by calcining the Ru/CoAl LDH, Rh/CoAl LDH, Pt/CoAl LDH and Pd/CoAl LDH in 5vol.% $H_2/N_2$ stream at 450 ℃ for 2 h, respectively.

**Characterizations**

The phase structure of the catalysts was determined by X-ray diffraction (XRD, Bruker D8) with a Cu-Kα source. Transmission electron microscopy (TEM) and

high-resolution TEM (HRTEM) images were recorded on a JEM-F200 instrument operated at 200 kV. High-angle annular dark-field scanning TEM (HAADF-STEM) image and energy-dispersive X-ray (EDX) elemental mappings were obtained using a JEOL JEM-ARM200F instrument with an aberration-corrected at 200 kV. UV-Vis diffuse reflectance spectra (DRS) were recorded on a UV-3600 spectrophotometer (Shimadzu). The specific surface area and pore size distribution were determined based on the Brunauer-Emmett-Teller (BET) and Barrett-Joyner-Halenda (BJH) methods, respectively. X-ray photoelectron spectroscopy (XPS) measurements were carried out on an ESCALAB 250XI instrument, and the data was calibrated with C1s peak at 284.8 eV. $H_2$ temperature-programmed reduction ($H_2$-TPR) and $CO_2$ temperature-programmed desorption ($CO_2$-TPD) measurements were undertaken on a Micromeritics AutoChem II 2920 instrument.

**Photoelectrochemical measurements**

Photocurrent response and electrochemical impedance spectra (EIS) were obtained on an electrochemical workstation (CHI-660E) with a three-electrode system in $Na_2SO_4$ aqueous solution using Pt wire and Ag/AgCl electrode as the counter electrode and reference electrode, respectively. A 300 W Xe lamp was used in the regular on-off cycled photocurrent test.

**Photo-thermal properties measurements**

The studied samples (50 mg of the catalyst was diluted into 1.2 g of quartz sand) were

placed in an alumina crucible and the powder was then irradiated by a 300 W Xe lamp (420-780 nm; 2 W $cm^{-2}$). The temperature changes were recorded every 20 seconds with a thermal imager (FLIR E8XT).

**Numerical calculations**

Numerical calculations based on finite element methods were performed using COMSOL Multiphysics software packages to simulate the electromagnetic field distribution on the nanomaterials. This method involves the discretization of Maxwell's equations in the space domain to simulate the electric field distributions at different positions. The incident power was set to be 2.0 W/$cm^2$ (420 nm), while the diameters of Ir nanoparticles and CoO were 2 nm and 10 nm, respectively. The permittivity of Ir and CoO was adopted from previous studies.[S1] In addition, the heat generation was calculated based on the steady-state heat transfer model. The thermal diffusion equation in the steady-state regime is as follows:

$$\nabla (-k\nabla T) = f$$

Where k is the thermal conductivity, T is the temperature and f is the power density of heat source. The thermal conductivity of air, Ir and CoO used in this study are 0.03, 147 and 9.17 W $m^{-1}$ $K^{-1}$, respectively.

**Photo-thermal catalytic performance evaluation**

The photo-thermal $CO_2$ hydrogenation reaction activity was evaluated in a continuous flow fixed-bed quartz tubular reactor at atmospheric pressure (Beijing China

Education Au-light Co., China, Figure S1). Typically, 50 mg of the catalyst was mixed with 1.2 g of quartz sands (ca. 0.42 mm in diameter) and then the mixture was packed in the reactor so as to avoid the flow choking caused by the agglomeration of the nanocatalysts during reaction process. A 1:4 mixture of $CO_2$ and $H_2$ with a total flow rate of 20 $mL\cdot min^{-1}$ was introduced into the filled reactor. The gaseous hourly space velocity (GHSV) applied was 24000 $cm^3\ h^{-1}\ g_{cat}^{-1}$. A 300 W Xe lamp (420-780 nm) was used to illuminate the catalyst through a quartz window from the side of the reactor and the area for irradiation was 5.76 $cm^2$ (2.4 cm×2.4 cm). The light intensity was measured of 2 W $cm^{-2}$ with a light power meter (CEL-NP2000). A thermocouple inserted into the quartz tube just underneath the catalyst bed monitored the catalyst temperature. The concentrations of $CO_2$ and products in the effluent gas were analyzed using an online gas chromatograph equipped with a flame ionization detector (FID) and a thermal conductivity detector (TCD) using $N_2$ as carrier gas.

The $CO_2$ conversion ($X_{CO2}$, %), products selectivity (S, %) and $CH_4$ production rate ($rCH_4$) were calculated according to the following equations:

$$X_{CO2}\ (\%) = \frac{n_{CO_2,\,in} - n_{CO_2,\,out}}{n_{CO_2,\,in}} \times 100$$

$$S_{CH4}\ (\%) = \frac{n_{CH_4}}{n_{CH_4}+n_{CO}+n_{CH_3OH}} \times 100$$

$$S_{CH3OH}\ (\%) = \frac{n_{CH_3OH}}{n_{CH_4}+n_{CO}+n_{CH_3OH}} \times 100$$

$$S_{CO}\ (\%) = 100\% - S_{CH4}\ (\%) - S_{CH3OH}\ (\%)$$

$$r(CH_4) = \frac{v_{CO_2} \times t \times X_{CO_2} \times S_{CH_4}}{V_m \times m}$$

Where $n_{CO2,\,in}$ and $n_{CO2,\,out}$ are the mole numbers of $CO_2$ in the inlet and outlet; $n_{CH4}$,

$n_{CO}$ and $n_{CH3OH}$ represent the mole numbers of $CH_4$, $CH_3OH$ and CO in the products.

***In situ* diffuse reflectance infrared Fourier transform spectroscopy (*In situ* DRIFTS) analysis**

*In situ* DRIFTS measurements were performed on a Bruker Vertex 70 spectrometer with a mercury-cadmium-telluride (MCT) detector. The DRIFTS reaction cell (Harrick) was equipped with $CaF_2$ windows and a heating cartridge. Prior to collecting the spectra, the sample was activated in 5% vol.% $H_2$/Ar at 300 ℃ for 30 min and then cooled to room temperature. Then the reaction cell was flushed with $N_2$ for 20 min and the spectra of samples were recorded as the background. Subsequently, a mixture of 15 vol.% $CO_2$, 30 vol.% $H_2$ and 55 vol.% Ar was introduced into the reaction cell (20 mL $min^{-1}$). The spectra were collected after the steady-state was reached. To investigate the effect of illumination on the reaction pathway and kinetics, the light was introduced by an optical fiber equipped with a 300 W Xe lamp (420-780 nm; 0.18 W $cm^{-2}$) into the reaction cell passing through the quartz window. The spectra were collected under light irradiation at 300 ℃.

**Density functional theory (DFT) calculations**

DFT were carried out using the Vienna Ab-initio Simulation Package (VASP) code. The generalized gradient approximation with the Perdew-Burke-Ernzerh (PBE) function was used to describe the correlation effects and electronic exchange. The wave functions were constructed from the expansion of plane waves with an energy cutoff of 450 eV. Gamma centered k-piont of 2×2×1 has been used for geometry

optimization. The consistence tolerances for the geometry optimization are set as 1.0 $\times 10^{-5}$ eV/atom for total energy and 0.05 eV/Å for force, respectively. In order to avoid the interaction between the two surfaces, a large vacuum gap of 15 Å has been selected in the periodically repeated slabs. The adsorption energy was calculated according to the standard formula:

$$E_{ads} = E_{Total} – E_{Cat} – E_{Molecular}$$

Where $E_{ads}$ represents the adsorption energy of $CO_2$ and $H_2$ molecules on the neutral or negatively charged Ir(111) surface, $E_{Total}$ represents the system total energy, $E_{Cat}$ represents the energy of catalyst and $E_{Molecular}$ represents the energy of a single $CO_2$ or $H_2$ molecule.

In free energies calculations, the entropic corrections and zero-point energy (ZPE) have been included. The free energy of species was calculated according to the standard formula:

$$\Delta G = E + \Delta ZPE + \Delta H – \Delta TS$$

where ZPE was the zero-point energy, ΔH was the integrated heat capacity, T was the temperature of product, and S was the entropy.

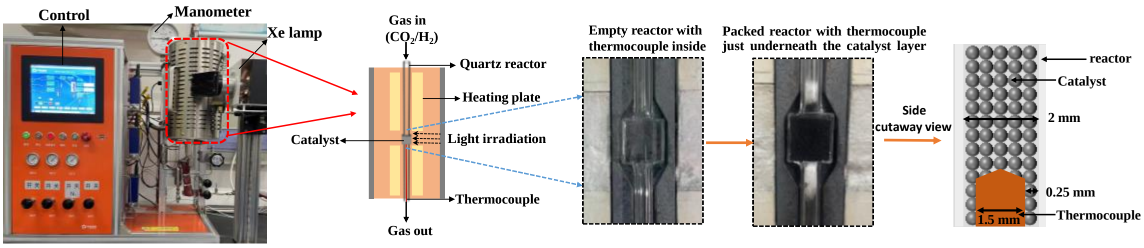


**Figure S1.** Digital image of equipment for the photo-thermal $CO_2$ hydrogenation and the schematic illustration of the reactor.

Supplementary discussion:

XRD patterns confirmed the successful synthesis of target materials (Figure S2a). In the XRD pattern of Ir nanoparticles, the diffraction peaks at 40.66°, 47.30°, 69.13°, 83.41 and 88.03° are indexed to the (111), (200), (220), (311) and (222) facets of fcc Ir (JCPDS No. 46-1044), respectively (Figure S2b). Moreover, the diffuse of the generated volatile gases including $CO_2$ and $H_2O$ during sample preparation process might be responsible for the formation of rough and porous surfaces of $Al_2O_3$ nanosheets (Figure S2c). UV-Vis DRS study indicated that 0.16%Ir-CoO/$Al_2O_3$ shows strong absorption across the UV-Vis-NIR region, hinting its high solar energy harnessing capacity (Figure S2d).

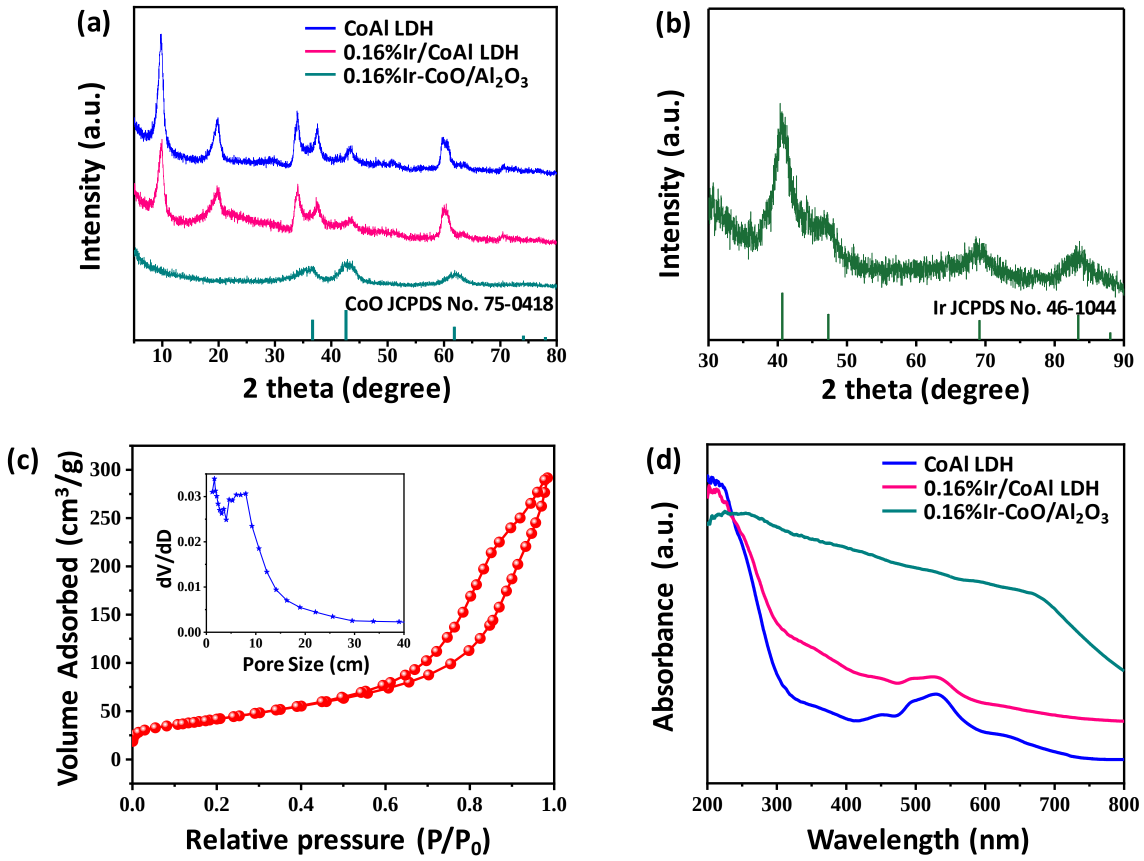


**Figure S2.** XRD patterns of (a) CoAl LDH, 0.16%Ir/CoAl LDH, 0.16%Ir-CoO/$Al_2O_3$ and (b) Ir nanoparticles. (c) Nitrogen adsorption-desorption isotherms and the corresponding pore-size distribution curves (inset) of 0.16%Ir-CoO/$Al_2O_3$. (d) UV-Vis DRS of CoAl LDH, 0.16%Ir/CoAl LDH and 0.16%Ir-CoO/$Al_2O_3$.

Supplementary discussion:

To investigate the band structure of CoO, XPS valence band spectrum was measured (Figure S3c). The measured valence band potential ($E_{VB\text{-}XPS}$) of CoO is 0.32 eV and therefore, $E_{VB\text{-}XPS}$ value relative to the standard hydrogen electrode ($E_{VB\text{-}NHE}$) is calculated to be 0.42 eV according to the formula: $E_{VB\text{-}NHE}=\Phi+E_{VB\text{-}XPS}-4.44$, where $\Phi$ is the electron work function of the instrument (4.54 eV).[S2] According to the $E_g$ results (Figure S3b) and valence band position, the conduction band potential ($E_{CB\text{-}NHE}$) of CoO is determined as -1.68 eV. The redox potentials of the relevant reactions for $CO_2$ reduction with respect to the estimated position of the CoO band edges versus NHE (pH=7) were shown in Figure S3d. Thus, the reduction of $CO_2$ into CO, $CH_4$ and $CH_3OH$ over CoO is energetically favorable.

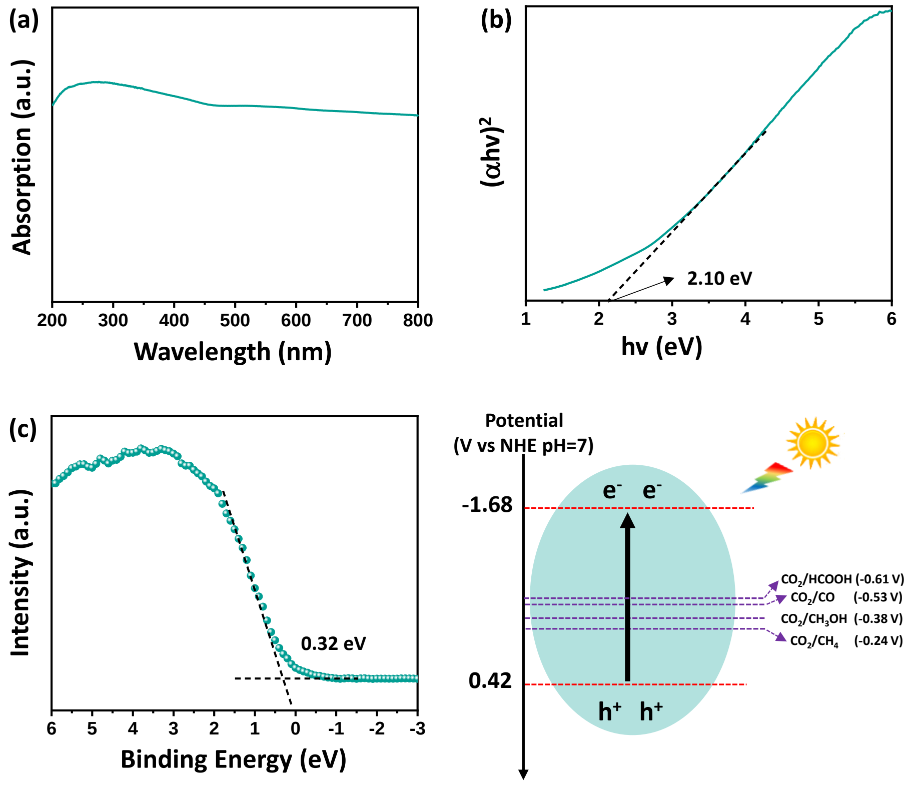

**Figure S3.** (a) UV-Vis DRS and (b) corresponding Tauc plot of CoO. (c) XPS valence band spectrum of CoO. (d) Schematic diagram of the estimated band positions of CoO relative to the reduction potentials of $CO_2$ to various products.

Supplementary discussion:

It can be revealed from the TEM images in Figure S4a-b that the spherical Ir nanoparticles of ca. 1.7 nm with narrow size distributions and CoAl LDH assembled with numerous nanosheets were successfully synthesized. For 0.16%Ir/CoAl LDH composite, it can be observed that Ir nanoparticles are uniformly dispersed on the surface of CoAl LDH, and the corresponding lattice spacing of 0.22 nm is assigned to Ir (111) plane (Figure S4c-d). EDX elemental mappings further showed the even distribution of Ir nanoparticles on CoAl LDH support (Figure S4e-i).

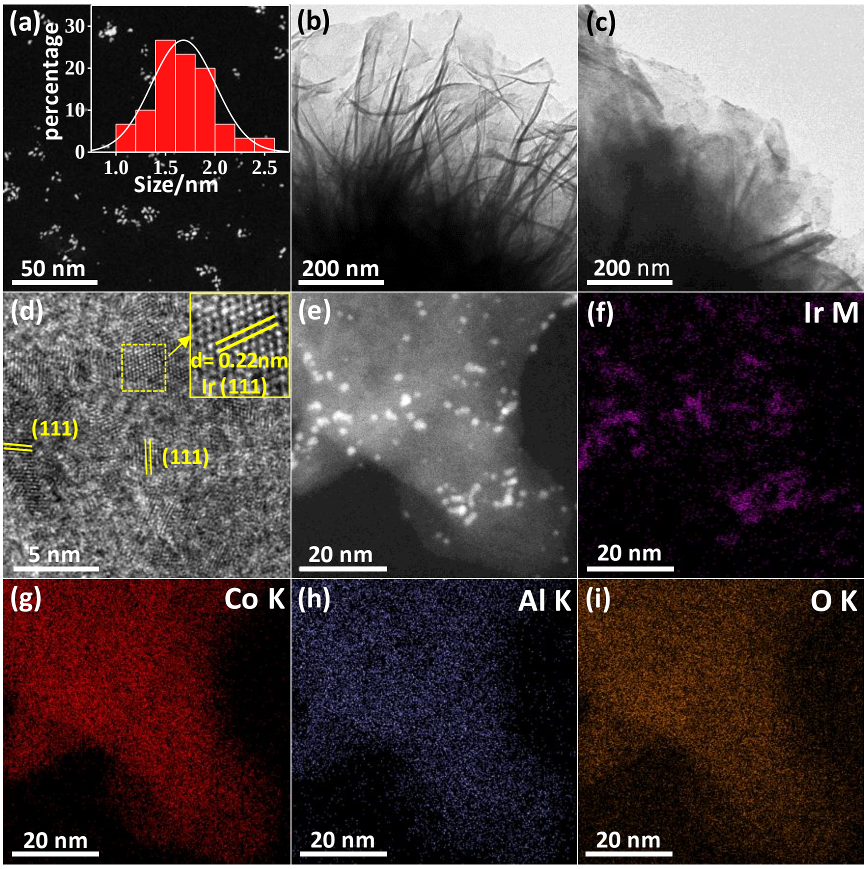

**Figure S4.** TEM images of (a) Ir nanoparticles, (b) CoAl LDH and (c) 0.16%Ir/CoAl LDH. (d) HRTEM image, (e) HAADF-STEM image and (e-i) corresponding EDX elemental mappings of 0.16%Ir/CoAl LDH.

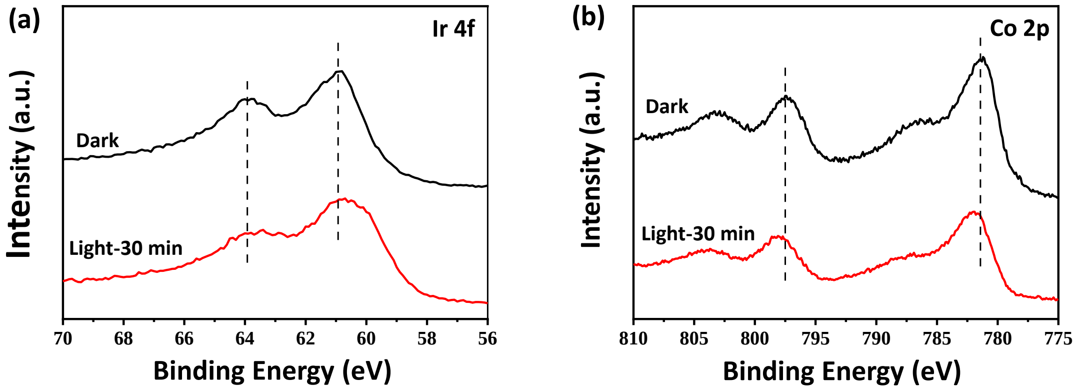


**Figure S5.** Ir 4f and Co 2p XPS spectra of 0.16%Ir-$CoO/Al_2O_3$ in dark and under light irradiation.

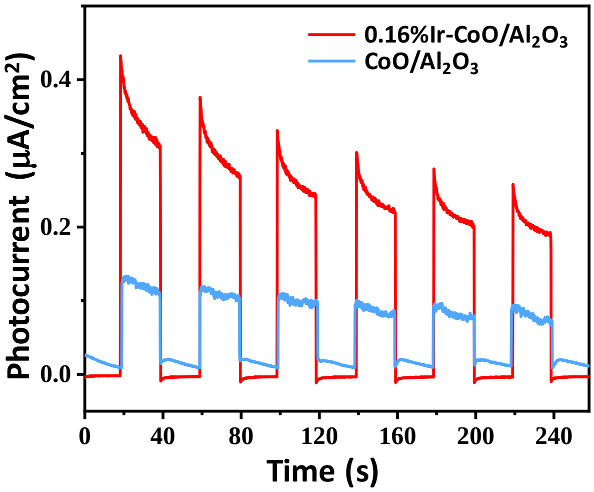


**Figure S6.** Photocurrent responses of $CoO/Al_2O_3$ and 0.16%Ir-$CoO/Al_2O_3$.

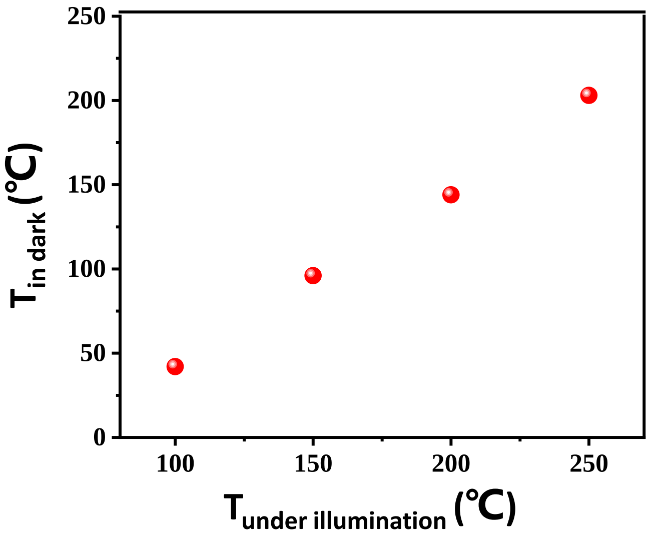


**Figure S7.** Temperature difference with/without light irradiation over 0.16%Ir-CoO/$Al_2O_3$ catalyst.

Supplementary discussion:

The 0.16%Ir/CoAl LDH shows a $CH_4$ production rate of 121 mmol $g^{-1}{}_{cat}$ $h^{-1}$ at 250 ℃ under light irradiation (Figure S8b), which is similar to 0.16%Ir-CoO/$Al_2O_3$ (128.9 mmol $g^{-1}{}_{cat}$ $h^{-1}$). Noteworthily, it is found that the selectivity to CO declines sharply whilst that of $CH_4$ progressively augments, along with the rise of temperatures and the highest $CH_4$ selectivity of 90% at 250 ℃. That is probably because of the structural topological transformation from Ir/CoAl LDH to Ir-CoO/$Al_2O_3$ with the increase of temperature in the reducing atmosphere and almost complete transformation into Ir-CoO/$Al_2O_3$ at 250 ℃ under light irradiation. The result can also be confirmed by the

XRD pattern of used 0.16%Ir/CoAl LDH. As shown in Figure S8a, the spent Ir/CoAl LDH catalyst shows diffraction characteristics of cubic CoO, indicating the structural transformation of Ir/CoAl LDH to Ir-CoO/$Al_2O_3$ during $CO_2$ methanation reaction. The result further demonstrates that the high $CH_4$ production rate and selectivity are attributed to the intimate interaction between Ir nanoparticles and CoO.

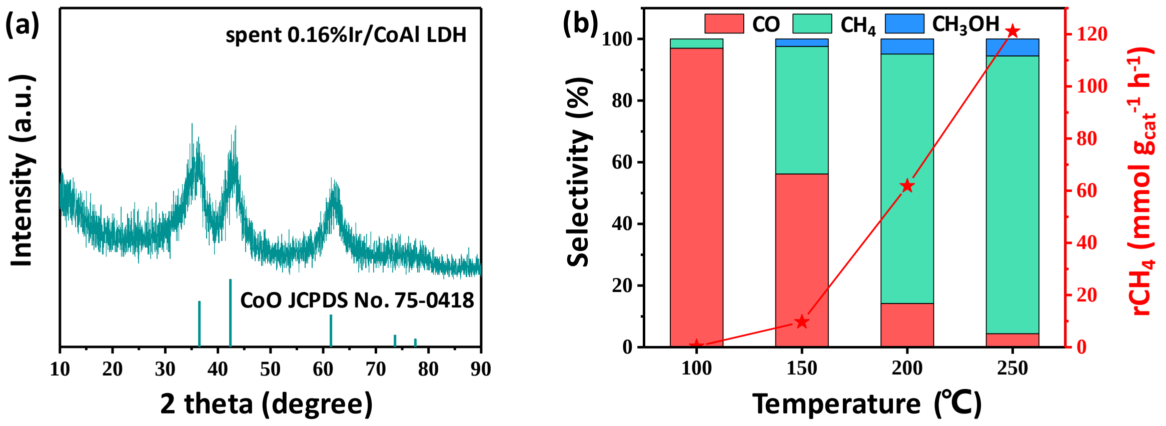


**Figure S8.** (a) XRD patterns of used 0.16%Ir/CoAl LDH. (b) Product selectivity and $CH_4$ yield rate of 0.16%Ir/CoAl LDH catalyst at different temperatures.

Supplementary discussion:

As shown in the TEM images (Figure S9a-d), Ru, Rh, Pd and Pt nanoparticles are uniformly dispersed on the surface of CoAl LDH, respectively. After being annealed at 450 °C in 5 vol.% $H_2/N_2$, the structure of these composites underwent a topological transformation, consistent with Ir/CoAl LDH sample and the corresponding TEM images are shown in Figure S9e-h. Besides, the XRD patterns show that the Ru/CoAl LDH, Rh/CoAl LDH, Pt/CoAl LDH and Pd/CoAl LDH composites transform into Ru-CoO/$Al_2O_3$, Rh-CoO/$Al_2O_3$, Pt-CoO/$Al_2O_3$ and Pd-Co/$Al_2O_3$ after heat treatment,

respectively (Figure S9i). In addition, the catalytic activity of Ru-CoO/$Al_2O_3$, Rh-CoO/$Al_2O_3$, Pt-CoO/$Al_2O_3$ and Pd-Co/$Al_2O_3$ catalysts also were evaluated. As shown in Figure S9j, Ru-CoO/$Al_2O_3$ catalyst exhibits a superb $CH_4$ production rate of 98.4 mmol $g_{cat}^{-1}$ $h^{-1}$ with selectivity up to 90.2%. Rh-CoO/$Al_2O_3$ catalyst shows a relatively low $CH_4$ production rate of 31.4 mmol $g_{cat}^{-1}$ $h^{-1}$ with 70% selectivity and the rate further decreased to 0.14 mmol $g_{cat}^{-1}$ $h^{-1}$ on Pt-CoO/$Al_2O_3$ catalyst. However, Pd-Co/$Al_2O_3$ exhibits no activity for $CO_2$ hydrogenation reaction, which could be related to the presence of Co nanoparticles. To verify this hypothesis, the catalytic activity of the Pd/CoAl LDH catalyst was studied (Figure S9k). It is worth noting that the yields of $CH_4$ and CO increase first and then decrease with the rise in temperatures and the production rates of $CH_4$ (5.7 mmol $g_{cat}^{-1}$ $h^{-1}$) and CO (14.9 mmol $g_{cat}^{-1}$ $h^{-1}$) peak at 200 ℃. This result could be attributed to the structural topological transformation from Pt/CoAl LDH to Pt-CoO/$Al_2O_3$ and then to Pt-Co/$Al_2O_3$ in the $CO_2$ hydrogenation reaction process with the temperature increasing. Therefore, we speculate that the enhanced catalytic performance is primarily attributed to the interaction between active metal nanoparticles and CoO.

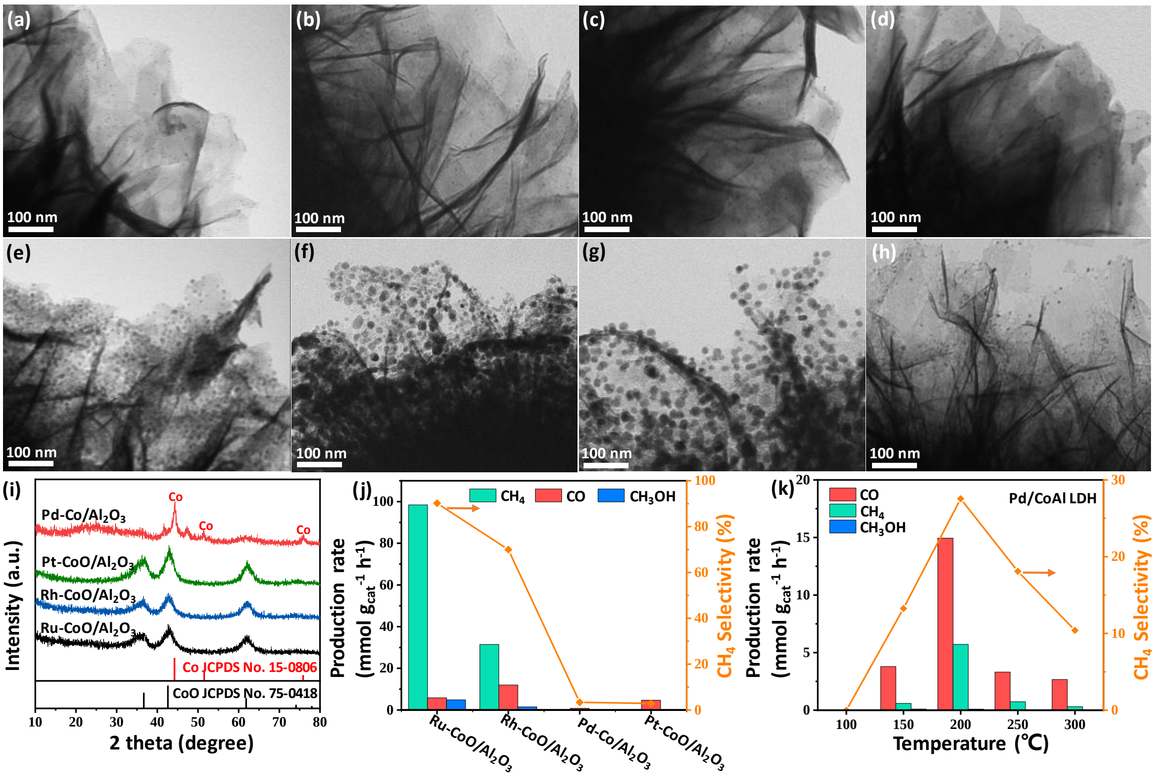


**Figure S9.** TEM images of (a) Ru/CoAl LDH, (b) Rh/CoAl LDH, (c) Pd/CoAl LDH, (d) Pt/CoAl LDH, (e) Ru-CoO/$Al_2O_3$, (f) Rh-CoO/$Al_2O_3$, (g) Pd-Co/$Al_2O_3$, and (h) Pt-CoO/$Al_2O_3$. (i) XRD pattern and (j) $CO_2$ hydrogenation performances of Ru-CoO/$Al_2O_3$, Rh-CoO/$Al_2O_3$, Pd-Co/$Al_2O_3$ and Pt-CoO/$Al_2O_3$ catalysts at 250 ℃ under light irradiation. (k) $CO_2$ hydrogenation performance of Pd/CoAl LDH as a function of temperatures under light irradiation.

Supplementary discussion:

The Arrhenius plot was plotted according to the formula: $\ln(k) = \ln(A) - (E_a/R)*(1/T)$, where k, R and T are rate constant, gas constant and absolute temperature, respectively. Therefore, the Arrhenius equation in dark and under light irradiation is obtained:

$$y = -2.595x + 9.828 \text{ (light)}$$

$$y = -2.979x + 8.732 \text{ (dark)}$$

The corresponding Arrhenius plot showed one-stage linear plots with activation energies of 40.80 kJ $mol^{-1}$ in dark and 38.49 kJ $mol^{-1}$ under light irradiation, elucidating that illumination could lead to the decrease in apparent activation energy (Figure S10b).

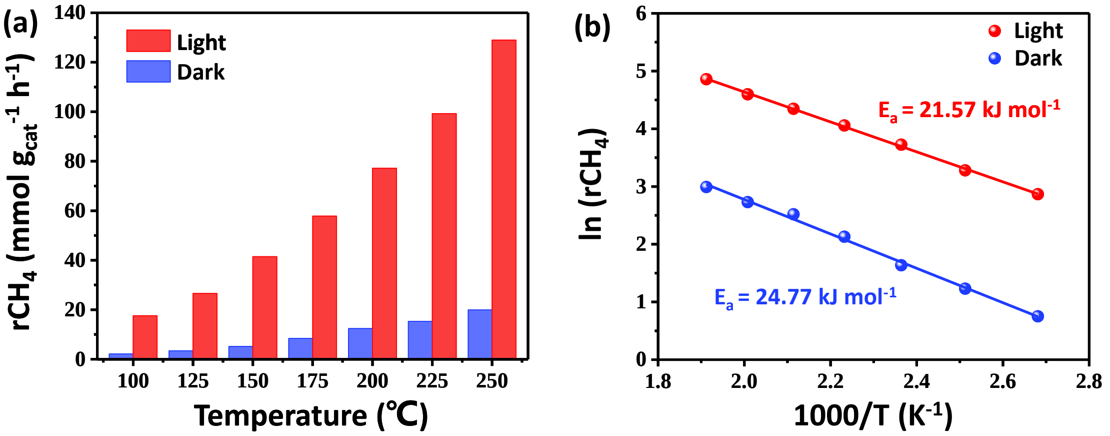


**Figure S10** (a) $CH_4$ production rate on 0.16%Ir-CoO/$Al_2O_3$ in dark and under light irradiation at different temperatures and (b) the corresponding Arrhenius plot.

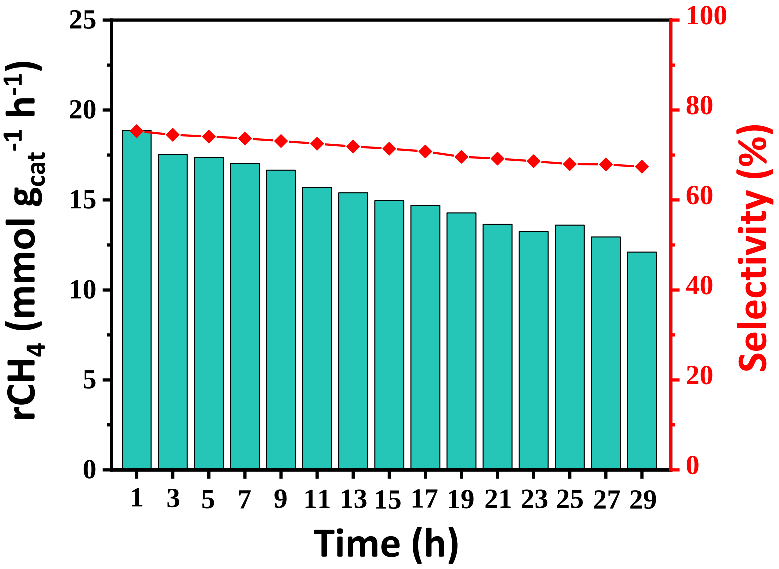


**Figure S11.** $CH_4$ yield rate and selectivity of 0.16%Ir-CoO/$Al_2O_3$ catalyst during

long-time reaction in dark.

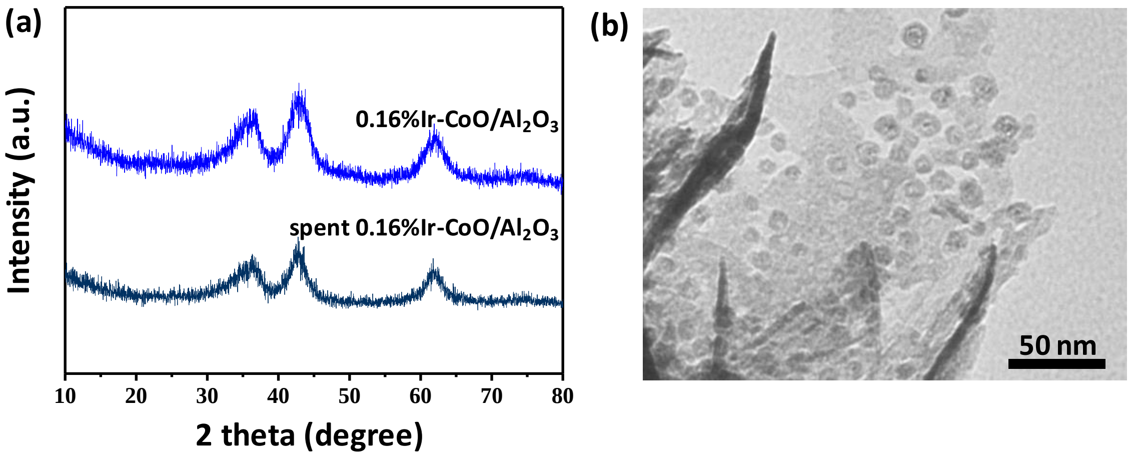


**Figure S12.** (a) XRD patterns of 0.16%Ir-CoO/$Al_2O_3$ before and after $CO_2$ hydrogenation reaction test. (b) TEM image of spent 0.16%Ir-CoO/$Al_2O_3$ catalyst.

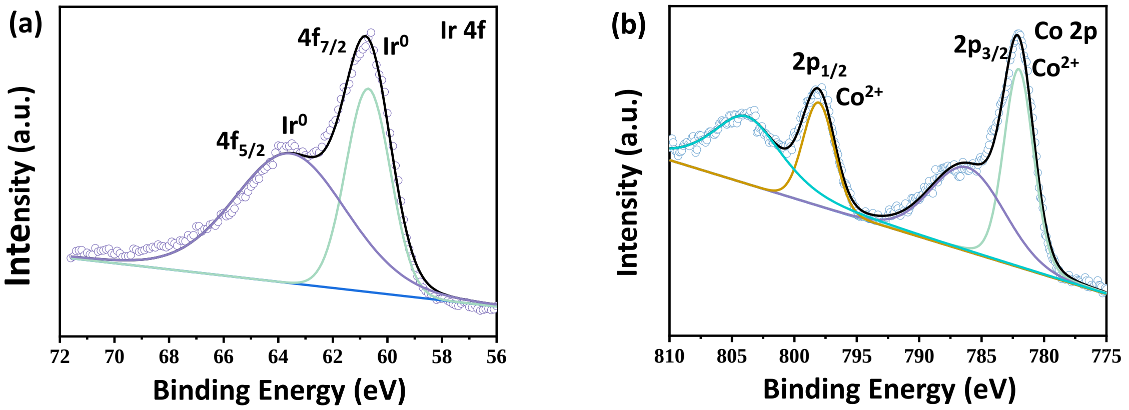


**Figure S13.** Ir 4f and Co 2p XPS spectra of 0.16%Ir-CoO/$Al_2O_3$ after $CO_2$ hydrogenation.

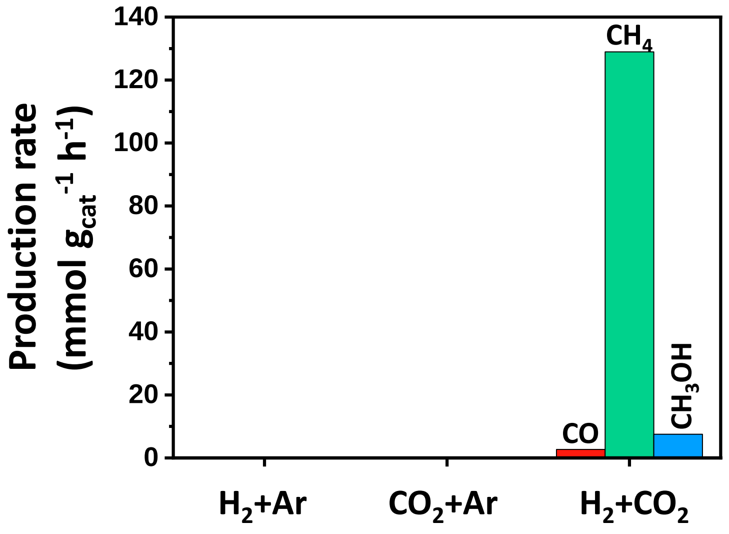


**Figure S14.** Production rate of CO, $CH_4$ and $CH_3OH$ over 0.16%Ir-CoO/$Al_2O_3$ catalyst using different reaction gases.

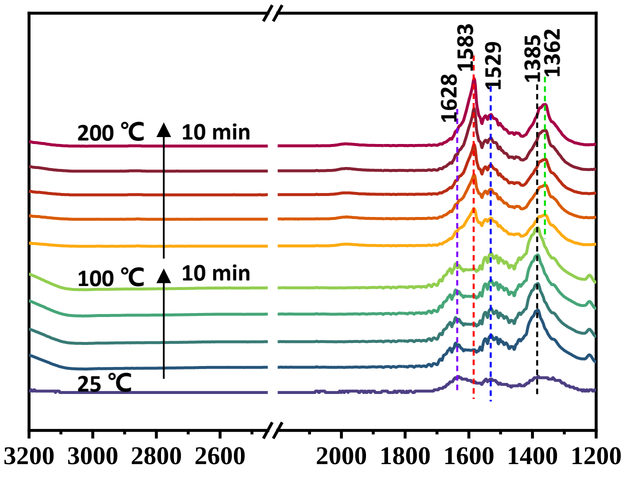


**Figure S15.** *In situ* DRIFTS spectra of 0.16%Ir-CoO/$Al_2O_3$ catalyst in the mixture of $H_2$ and $CO_2$ (2:1) from 25 ℃ to 200 ℃ in dark.

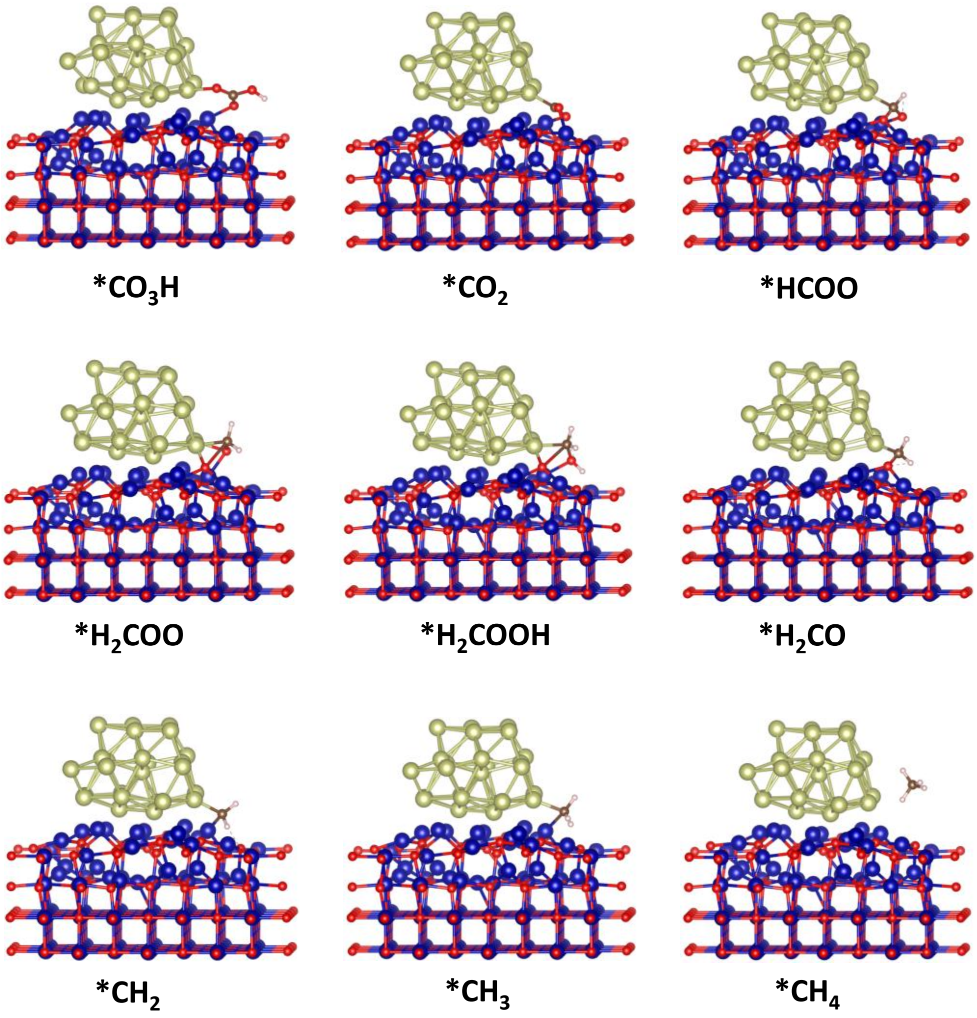


**Figure S16.** The optimized geometric structure of involved intermediates.

**Table S1.** The loading of Ir nanoparticles was measured by ICP-OES.

| **Sample** | **Ir loading (wt.%)** |
|---|---|
| 0.03%Ir-CoO/$Al_2O_3$ | 0.03 |
| 0.07%Ir-CoO/$Al_2O_3$ | 0.07 |
| 0.16%Ir-CoO/$Al_2O_3$ | 0.16 |
| 0.23%Ir-CoO/$Al_2O_3$ | 0.23 |
| Ir-CoO | 0.25 |

| | |
|---|---|
| Ir/$Al_2O_3$ | 0.17 |
| Ir/CoO/$Al_2O_3$ | 0.19 |
| Ru-CoO/$Al_2O_3$ | 0.31 |
| Rh-CoO/$Al_2O_3$ | 0.13 |
| Pt-CoO/$Al_2O_3$ | 0.24 |
| Pd-Co/$Al_2O_3$ | 0.34 |

**Table S2.** The comparison of catalytic activities with other reported catalysts for photo-thermal catalytic $CO_2$ methanation.

| Catalysts | Light source | Temperature (℃) | Pressure (MPa) | $H_2/CO_2$ ratio | $CH_4$ rate (mmol $g_{cat}^{-1}$ $h^{-1}$) | Selectivity (%) | Ref. |
|---|---|---|---|---|---|---|---|
| Ir-CoO/$Al_2O_3$ | 300 W Xe lamp<br>2 W/cm$^2$, 420-780 | 250 | 0.1 | 4:1 | 128.9 | 92 | This work |
| Cu/ZnO | 300 W Xe lamp<br>2 W/cm$^2$, 420-780 | 300 | 0.1 | 4:1 | 0.052 | 100 | [S3] |
| $Co_7Cu_1Mn_1O_x$ (200) | 300 W Xe lamp<br>0.23 W/cm$^2$, 300-1100 | 200 | 0.1 | 3:1 | 14.5 | 85.3 | [S4] |
| Ir@UiO-66 | 300 W Xe lamp<br>2.3 W/cm$^2$, 200-1100 | 250 | 0.1 | 4:1 | 19.9 | 95 | [3c] |
| Ru/$TiO_2$ | 300 W Xe lamp<br>0.1 W/cm$^2$, 300-1100 | 250 | 0.1 | 3:1 | 46.15 | - | [7a] |
| Au@AuRu/g-$C_3N_4$ | 300 W Xe lamp<br>1.1 W/cm$^2$, Full | 150 | 1 | 3:1 | 0.103 | 98.4 | [S5] |
| $RuO_2$/STO | 300 W Xe lamp<br>0.1 W/cm$^2$, UV-Vis | 150 | 0.13 | batch | 14.6 | 89.5 | [S6] |
| Rh/$TiO_2$ | LED<br>1 W/cm$^2$, 365 | 250 | / | 3:1 | 88 | 98 | [S7] |
| $RuO_2$@MIL-125(Ti)-$NH_2$ | 150 W Hg-Xe lamp<br>0.1 W/cm$^2$, 420-780 | 200 | 0.13 | 4:1 | 0.84 | | [S8] |
| $Ag_{24}$Au/meso-$Co_3O_4$ | 300 W Xe lamp<br>~0.2 W/cm$^2$, 350-780 | 240<br>300 | 1.5 | 3:1 | 23<br>103 | 61<br>77 | [S9] |

**Table S3.** Calculated bond lengths and adsorption energy of adsorbed $H_2$ on neutral and negatively charged Ir(111) surface.

| Surface charge | Bond length (Å) | | | Adsorption energy (eV) |
|---|---|---|---|---|
| | H-H | Ir-H1 | Ir-H2 | |
| [Ir(111)] | 1.8396 | 1.6120 | 1.6173 | -0.7613 |
| $[Ir(111)]^-$ | 2.0289 | 1.5938 | 1.6393 | -1.0266 |

**References:**


[S1] a) S. S. A. Seo, K. W. Kim, T. W. Noh, M. Y. Kim, S. Shin, W. S. Choi, *Phys. Rev. B* **2006**, *74*, 205117; b) K. V. Rao, A. Smakula, *J. Appl. Phys.* **1965**, *36*, 2031-2038.

[S2] P. Gao, Z. Cui, X. Liu, Y. Wu, Q. Zhang, Z. Wang, Z. Zeng, H. Cheng, Y. Liu, Q. Li, B. Huang, P. Wang, *Chem. Eur. J.* **2022**, *28*, e202201095.

[S3] X. He, M. Liu, Z. Liang, Z. Wang, P. Wang, Y. Liu, H. Cheng, Y. Dai, Z. Zheng, B, Huang, *J. Solid State Chem.* **2021**, *298*, 122113.

[S4] Z. He, Z. Li, Z. Wang, K. Wang, Y. Sun, S. Wang, W. Wang, Y. Yang, Z. Liu, *Green Chem.* **2021**, *23*, 5775.

[S5] X. Zhang, H. Liu, Y. Wang, S. Yang, Q. Chen, Z. Zhao, Y. Yang, Q. Kuang, Z. Xie, *Chem. Eng. J.* **2022**, *443*, 136482.

[S6] D. Mateo, J. Albero, H. Garcia, *Joule* **2019**, *3*, 1949.

[S7] X. Zhang, X. Li, M. E. Reish, D. Zhang, N. Q. Su, Y. Gutiérrez, F. Moreno, W. Yang, H. O. Everitt, J. Liu, *Nano Lett.* **2018**, *18*, 1714.

[S8] M. Cabrero-Antonino, B. Ferrer, H.G. Baldoví, S. Navalón, *Chem. Eng. J.* **2022**, *445*, 136426.

[S9] Y. Xiong, X. Liu, Y. Hu, D. Gu, M. Jiang, Z. Tie, Z. Jin, *Nano Res.* **2022**, *15*, 4965-4972.
